# Supplementary material for: Dividing attention during the Timed Up and Go enhances associations of several subtask performances with MCI and cognition
Source: PLoS One. 2022 Aug 3;17(8):e0269398. doi: 10.1371/journal.pone.0269398 (PMC9348700; doi:10.1371/journal.pone.0269398)
Supplement: S1 Table — Neuropsychological assessments used to compute composite scores of cognitive abilities across attentional and executive control domains. As in our prior publications, composite scores were constructed by averaging across centered and scaled test scores, relative to the appropriate parent study baseline. (DOCX) [file pone.0269398.s001.docx]

**S1 Table**

| **Cognitive Domain** | **Test** | **Description** |
| --- | --- | --- |
| **Attention** | **Digit span forward** | Digit span forward is a test in which sequences of increasing length are read to participants, one at a time. Participants are then asked to repeat each sequence. Testing stops after two consecutive errors at a given sequence length. The primary measure of performance is the number of digit sequences correctly recalled. |
|  | **Number comparison** | Participants are presented with pairs of three- to ten-digit sequences. Some of the pairs are exactly the same while others do not match. Participants are asked to identify pairs as “same” or “different” with a 90-second time limit. Each correct answer is scored. |
|  | **Stroop word reading** | The Stroop word test is a measure of executive functioning and capacity to direct attention. Participants are asked to read aloud a list of words as quickly as they can, within a 30 second limit. The score is the number of words read correctly. |
| **Executive Function** | **Digit ordering** | The digit ordering test is modified from procedures used by Cooper, Sagar, Jordan, Harvey, and Sullivan (1991). A series of numbers are read aloud to the participants, one series at a time. After each series, participants are asked to order the digits in the series from smallest number to largest number. Each correct answer is scored. The test is administered from 2- to 8- digit length pairs. If both pairs of a certain length are not ordered properly, testing stops. |
|  | **Stroop color naming** | The Stroop color test is a measure of executive functioning and capacity to direct attention. Participants are asked to name aloud the color of ink that each word in the list is printed in as quickly as they can. The score is the number of colors correctly named within a 30 second limit. |
|  | **Category fluency** | Category fluency is a modified version of the CERAD verbal fluency measure (Morris et al., 1989). Participants are asked to generate exemplars from each of 2 categories (animals, fruits and vegetables) within a 60-second time limit per category. This variable is the sum of unique exemplars generated from both categories. If one of the category scores is missing, the remaining valid score is multiplied by 2. |
