# Supplementary material for: Dividing attention during the Timed Up and Go enhances associations of several subtask performances with MCI and cognition
Source: PLoS One. 2022 Aug 3;17(8):e0269398. doi: 10.1371/journal.pone.0269398 (PMC9348700; doi:10.1371/journal.pone.0269398)
Supplement: S2 Table — Measures extracted from each TUG performance were summarized as nine mobility scores to quantify four TUG subtasks. Some measures were flipped according to factor direction (e.g., AP jerk), denoted by a -1 multiplier. (DOCX) [file pone.0269398.s002.docx]

**S2 Table**

| **Subtask** | **Mobility Score** | **Measure** | **Description** |
| --- | --- | --- | --- |
| **Sit to Stand** | Complexity | Pitch SD (deg/s) | Standard deviation of pitch (mediolateral [ML] rotation) |
|  |  | Pitch jerk (deg/s^2^) | Slope during the transition interval (ML axis) |
|  |  | AP acceleration SD (ft/s^2^) | Standard deviation of anterior-posterior (AP) axis acceleration |
|  |  | -1*AP jerk (ft/s^3^) | Slope during the transition interval (AP axis) |
|  | Duration | Pitch duration (s) | Duration of transition interval: ML axis |
|  |  | AP duration (s) | Duration of transition interval: AP axis |
| **Walk** | Pace | Walking speed (ft/s) | Distance/time to complete subtask |
|  |  | Step length (ft) | Distance/number of steps |
|  | Cadence | Cadence (steps/min) | Number of steps/min |
|  | Variability | Step time CV (%) | COV of step time (std/mean step time x 100) |
|  | Regularity | Stride regularity | Autocorrelation of the signal time series |
| **Turn** | Magnitude | Yaw rate (deg/s) | Amplitude of angular velocity (rotation) around the vertical axis |
| **Stand to Sit** | Descent Control | Pitch SD (deg/s) | Standard deviation of pitch |
|  |  | AP acceleration SD (ft/s^2^) | Standard deviation of AP axis acceleration |
|  | Smoothness | Pitch jerk (deg/s^2^) | Slope during the transition interval (ML axis) |
|  |  | -1*Pitch duration (s) | Duration of transition interval: ML axis |
|  |  | AP jerk (ft/s^2^) | Slope during the transition interval (AP) |
|  |  | -1*AP duration (s) | Duration of transition interval: AP axis |
