# Supplementary material for: Dividing attention during the Timed Up and Go enhances associations of several subtask performances with MCI and cognition
Source: PLoS One. 2022 Aug 3;17(8):e0269398. doi: 10.1371/journal.pone.0269398 (PMC9348700; doi:10.1371/journal.pone.0269398)
Supplement: S3 Table — Univariate and forward selection linear regression results for the five cognitive domains traditionally used to construct global cognition. Models feature TUG performances, adjusted for age, sex, race (Black versus White), and education. Please note that β>0.10 has significance p<0.005, while β>0.133 has significance p < .0001. (DOCX) [file pone.0269398.s003.docx]

| **S3 Table** | | | **Episodic Mem.** | | **Semantic Mem.** | | **Working Mem.** | | **Perc. Speed** | | **Visuospatial** | |
| --- | --- | --- | --- | --- | --- | --- | --- | --- | --- | --- | --- | --- |
|  | **Subtask** | **Variable** | Ind R | FS R^2^ | Ind R | FS R^2^ | Ind R | FS R^2^ | Ind R | FS R^2^ | Ind R | FS R^2^ |
|  | *Overall* | *Duration* | -0.09 | - | -.15 | .01 | -.12 | - | -.22 | .005 | -.11 | - |
| **Quiet** | Stand | Complexity | ns | - | ns | - | ns | - | ns | - | ns | - |
|  |  | Duration | -.09 | - | -.09 | - | -.09 | - | -.10 | .0065 | ns | - |
|  | Walk | Pace | .12 | .03 | .15 | - | .16 | .04 | .27 | .08 | .14 | .035 |
|  |  | Cadence | 0.09 | - | .13 | - | .10 | - | .20 | - | .09 | - |
|  |  | Regularity | .11 | .006 | .14 | - | .11 | - | .20 | - | .09 | - |
|  |  | Variability | -.09 | - | -.19 | 0.04 | -.13 | .0035 | -.20 | - | -.12 | .0034 |
|  | Turn | Magnitude | 0.07 | - | .10 | - | .09 | - | .23 | - | .11 | - |
|  | Sit | Control | ns | - | ns | - | .09 | - | .16 | - | .09 | - |
|  |  | Smoothness | ns | - | ns | - | ns | - | .09 | - | ns | - |
|  | *Overall* | *Duration* | -.22 | .10 | -.21 | .08 | -.21 | - | -.29 | .01 | -.16 | .049 |
| **Dual Task** | Stand | Complexity | 0.07 | - | 0.08 | - | ns | - | .13 | - | ns | - |
|  |  | Duration | -.11 | - | -.12 | - | -.09 | - | -.16 | - | ns | - |
|  | Walk | Pace | .25 | .005 | .21 | .0095 | .24 | .08 | .31 | .11 | .17 | .0048 |
|  |  | Cadence | .21 | - | .18 | - | .17 | - | .22 | - | .13 | - |
|  |  | Regularity | .24 | - | .18 | - | .19 | - | .25 | - | .15 | - |
|  |  | Variability | -.22 | .01 | -.19 | - | -.18 | - | -.23 | .0028 | -.12 | - |
|  | Turn | Magnitude | .14 | - | .14 | - | .13 | - | .26 | .0025 | .12 | - |
|  | Sit | Control | .11 | - | .11 | - | .12 | - | .19 | - | .13 | - |
|  |  | Smoothness | ns | - | ns | - | ns | - | .09 | - | ns | .0031 |
|  | *Overall* | *Duration* | -.20 | 0.04 | - | - | -.14 | .02 | -.12 | .003 | ns | - |
| **Cost** | Stand | Complexity | ns | - | ns | - | ns | - | ns | - | ns | - |
|  |  | Duration | ns | - | ns | - | ns | - | ns | - | ns | - |
|  | Walk | Pace | .20 | - | ns | .011 | .14 | - | .10 | - | ns | - |
|  |  | Cadence | .18 | - | .11 | - | .13 | - | ns | - | ns | - |
|  |  | Regularity | .17 | .005 | 0.10 | - | .14 | .005 | .12 | .0124 | ns | .0065 |
|  |  | Variability | -.14 | - | ns | - | ns | - | ns | - | ns | - |
|  | Turn | Magnitude | ns | - | ns | - | ns | - | ns | - | ns | - |
|  | Sit | Control | ns | - | ns | - | ns | - | ns | - | ns | - |
|  |  | Smoothness | ns | - | ns | - | ns | - | ns | - | ns | - |
