# Supplementary material for: Dividing attention during the Timed Up and Go enhances associations of several subtask performances with MCI and cognition
Source: PLoS One. 2022 Aug 3;17(8):e0269398. doi: 10.1371/journal.pone.0269398 (PMC9348700; doi:10.1371/journal.pone.0269398)
Supplement: S4 Table — Individual metrics used to construct the nine mobility scores, as collected during the normal and dual-task Timed Up and Go. (DOCX) [file pone.0269398.s004.docx]

**S4 Table**

|  |  | **TUG** | **DT TUG** | **DT TUG - TUG** | | |
| --- | --- | --- | --- | --- | --- | --- |
|  | *Metric* | Mean (SD) | Mean (SD) | Mean (SD) | Cohen’s *d* | prob(t) |
| **Sit to Stand** | |  |  |  |  |  |
|  | Pitch SD (deg/s) | 57 (27) | 54 (24) | -3 (24) | -0.12 | <.001 |
|  | Pitch jerk (deg/s^2^) | 301 (345) | 256 (303) | -45 (335) | -0.13 | <.001 |
|  | AP acceleration SD (ft/s^2^) | 10.4 (4.1) | 10 (3.3) | -0.4 (3.6) | -0.098 | .009 |
|  | -1*AP jerk (ft/s^3^) | -56 (57) | -48 (44) | 8 (52) | 0.15 | <.001 |
|  | Pitch duration (s) | 0.8 (0.4) | 0.89 (0.4) | 0.05 (0.3) | 0.17 | <.001 |
|  | AP duration (s) | 0.92 (0.8) | 0.98 (0.7) | 0.06 (0.5) | 0.12 | .001 |
| **Walk** | |  |  |  |  |  |
|  | Walking speed (ft/s) | 2.5 (0.9) | 1.7 (0.8) | -0.8 (0.7) | -1.16 | <.001 |
|  | Step length (ft) | 1.5 (0.5) | 1.2 (0.4) | -0.3 (0.3) | -0.97 | <.001 |
|  | Cadence (steps/min) | 98 (13) | 83 (16) | -15 (13) | -1.09 | <.001 |
|  | Step time CV (%) | 13 (1) | 18 (1) | 5 (1) | 0.58 | <.001 |
|  | Stride regularity | 0.8 (0.1) | 0.7 (0.2) | -0.1 (0.18) | -0.73 | <.001 |
| **Turn** | |  |  |  |  |  |
|  | Yaw rate (deg/s) | 145 (42) | 131 (40) | -14 (26) | -0.54 | <.001 |
| **Stand to Sit** | |  |  |  |  |  |
|  | Pitch jerk (deg/s^2^) | 195 (106) | 179 (100) | -16 (92) | -0.18 | <.001 |
|  | -1*Pitch duration (s) | 0.97 (0.3) | 1.07 (0.4) | 0.10 (0.36) | 0.29 | <.001 |
|  | AP jerk (ft/s^2^) | 45 (18) | 44 (18) | -1 (16) | -0.06 | .088 |
|  | -1*AP duration (s) | 0.89 (0.4) | 0.94 (0.4) | 0.05 (0.4) | 0.13 | <.001 |
|  | Pitch SD (deg/s) | 48.7 (15) | 48.2 (15) | -0.5 (11) | -0.05 | .196 |
|  | AP acceleration SD (ft/s^2^) | 10.5 (2.7) | 10.7 (2.6) | 0.2 (2) | 0.08 | .023 |
